# Kaempferide Protects against Myocardial Ischemia/Reperfusion Injury through Activation of the PI3K/Akt/GSK-3*β* Pathway

**DOI:** 10.1155/2017/5278218

**Published:** 2017-08-27

**Authors:** Dong Wang, Xinjie Zhang, Defang Li, Wenjin Hao, Fanqing Meng, Bo Wang, Jichun Han, Qiusheng Zheng

**Affiliations:** ^1^Department of Cardiac Surgery, Shandong Provincial Qianfoshan Hospital, Shandong University, Jinan 250014, China; ^2^Binzhou Medical University, Yantai, Shandong 264003, China; ^3^Weifang Medical University, Weifang, Shandong 261053, China

## Abstract

The aim of this study is to investigate both the efficacy and mechanism of action of kaempferide (Kae) as a therapy for the treatment of cardiovascular disease. A rat model of myocardial ischemia/reperfusion (I/R) injury was established by ligation of the left anterior descending coronary artery for 30 min followed by a 2 h perfusion. In our study, we show that Kae remarkably improved cardiac function, alleviated myocardial injury via a decrease in myocardial enzyme levels, and attenuated myocardial infarct size in a dose-dependent manner. In addition, preconditioning treatment with Kae was found to significantly decrease serum TNF-*α*, IL-6, C-reactive protein (CRP), MDA, and ROS levels, while it was found to increase serum levels of SOD. Nuclear factor erythroid 2-related factor 2 (Nrf2) and cleaved caspase-3 expression levels were observed to be downregulated, while phospho-Akt (p-Akt) and phospho-glycogen synthase kinase-3*β* (p-GSK-3*β*) expression levels were upregulated. However, cotreatment with LY294002 (a PI3K inhibitor) or TDZD-8 (a GSK-3*β* inhibitor) was found to abolish the above cardioprotective effects observed with the Kae treatment. The data presented in this study provides evidence that Kae attenuates I/R-induced myocardial injury through inhibition of the Nrf2 and cleaved caspase-3 signaling pathways via a PI3K/Akt/GSK 3*β*-dependent mechanism.

## 1. Introduction

Acute myocardial infarction (AMI) is a prevalent disease associated with high morbidity and mortality rates [1]. In China alone, over 700,000 people reportedly die from this disease every year [2]. Currently, the most successful therapeutic strategy involves the pharmacological or mechanical restoration of coronary blood flow to preserve viable myocardium following AMI [3]. While reperfusion therapy has brought new hope for the reduction of myocardial damage, reperfusion itself has been shown to potentially induce a localized oxidative burst and a regional inflammatory response, resulting in cell damage and, in some cases, death. This pathophysiologic process has been defined in the field as an ischemia/reperfusion injury (I/R injury) [4]. In order to improve clinical outcomes in the case of AMI, it is critical to develop novel pharmacological agents for the prevention of myocardial I/R injury.

I/R injury is an intricate process involving numerous mechanisms. Oxidative damage has been demonstrated to play an important role in I/R injury progression [5]. In addition, antiapoptosis and anti-inflammation mechanisms have been reported to play protective roles for the heart in the case of I/R injury. This provides further evidence that apoptosis, inflammation, and oxidative injury all play a role in I/R injury [6, 7]. Therefore, the modulation of apoptosis, inflammation, oxidative damage, and related cascade responses is considered crucial therapeutic strategies for the treatment of cardiovascular I/R disease.

Phosphoinositide 3-kinases (PI3K) and their downstream target, protein kinase B (Akt), have been shown to be involved in the regulation of oxidation, inflammatory responses, and apoptosis [8–10]. Previous studies have indicated that the PI3K/Akt/GSK-3*β* signaling pathway may function as an endogenous negative feedback regulator that generates a compensatory mechanism to limit proinflammatory and apoptotic events in response to harmful stimuli [11, 12]. Activation of PI3K/Akt/GSK3*β*-dependent signaling has been demonstrated to result in the attenuation of myocardial I/R injury [13].

Flavonoids possess unique antioxidant properties and other pharmacological activities that may be relevant in protecting the heart from I/R injury; studies have also found that many flavonoids can protect against myocardial I/R injury [14, 15]. Kaempferide (3,5,7-trihydroxy-4′-methoxyflavone, Kae) is a naturally occurring flavonoid that is isolated from the roots of *Alpinia officinarum* (lesser galangal) [16]. Like other flavonoids, Kae also has a very good antioxidant properties and Kae can effectively reduce 1,1-diphenyl-2-picrylhydrazyl (DPPH) [17]. Studies have shown that Kae has anticancer [18, 19] and antihypertension effects [20]. However, little is known regarding the mechanism of action of Kae as a therapeutic agent for the treatment of cardiovascular disease. Thus, in this current study, we aim to investigate (1) whether Kae protects rat myocardium from I/R injury in an *in vivo* rat model and (2) the possible role of PI3K/Akt/GSK-3*β* signaling in the protective effects of Kae against myocardial I/R injury.

## 2. Material and Methods

### 2.1. Ethics Statement

The animals were handled, and all procedures were performed in accordance with the regulations of the Guide for the Care and Use of Laboratory Animals, approved by the Animal Care and Use Committee of Shandong University (publication number SYXK (Lu) 20130001, revised 2013).

### 2.2. Test Compounds, Chemicals, and Reagents

Kaempferide (purity ≥ 98%) was purchased from Chengdu Must Bio-Technology Co. LTD. (Chengdu, China). 1,1,3,3-Tetramethoxypropane was obtained from Fluka Chemical Co. (Ronkonkoma, NY). 2,3,5-Triphenyltetrazolium chloride, oxidized glutathione, and reduced glutathione were purchased from Sigma Chemical Co. (St. Louis, MO). The creatine kinase (CK) kits, lactate dehydrogenase (LDH) kits, superoxide dismutase (SOD) kits, malondialdehyde (MDA) kits, reactive oxygen species (ROS) kits, C-reactive protein (CRP) kits, interleukin-6 (IL-6) kits, and tumor necrosis factor-*α* (TNF-*α*) kits were purchased from Tsz Biosciences (Greater Boston, USA). All other chemicals and reagents were of analytical grade.

### 2.3. Animals

Adult Sprague Dawley (SD) rats were obtained from Jinan Pengyue Experimental Animal Breeding Co. Ltd. (license number: SCXK (lu) 2014-0007). Animals were between 250–300 g in weight and were housed in a room at 22–25°C, with a relative humidity between 50–60%, and a 12 h light/12 h dark cycle. All experimental protocols were approved by the Institutional Animal Care and Use Committee of Shandong University. Methods were carried out in accordance with the approved guidelines.

### 2.4. Ischemia/Reperfusion (I/R) Rat Model

We performed coronary artery ligation (CAL) to induce I/R injury in SD rats. Our modeling method mainly refers to the reference [21] and makes corresponding improvements. In brief, rats were anesthetized using 10% chloral hydrate (300 mg/kg body weight) via intraperitoneal injection and were ventilated with a HX-300S animal respirator (Chengdu Technology & Market Co. Ltd., Chengdu, China) (tidal volume, 6–8 ml/kg; ventilator frequency, 80 breaths/min). The heart was exposed via a left thoracotomy, and the left anterior descending (LAD) coronary artery was ligated for 30 min followed by a 2 h reperfusion. Muscle tone and tail/pedal withdrawal response were used to monitor anesthesia adequacy. At the completion of the experiment, rats were sacrificed by the administration of pentobarbital sodium (200 mg/kg) via intraperitoneal injection and phlebotomy.

### 2.5. Experimental Groups

Rats were randomly divided into 7 groups: (1) sham group, rats underwent sham surgery; (2) I/R group, rats were subjected to a 30 min LAD coronary artery ligation followed by a 2 h reperfusion; (3) L-Kae + I/R group, rats were injected with a low dose of Kae (L-Kae; 0.1 mg/kg body weight) 30 min prior to I/R, then subjected to a 30 min LAD coronary artery ligation followed by a 2 h reperfusion; (4) M-Kae + I/R group, rats were injected with a moderate dose of Kae (M-Kae; 0.3 mg/kg body weight) 30 min prior to I/R, then subjected to a 30 min LAD coronary artery ligation followed by a 2 h reperfusion; (5) H-Kae + I/R group, rats were injected with a high dose of Kae (H-Kae; 1 mg/kg body weight) 30 min prior to I/R, then subjected to a 30 min LAD coronary artery ligation followed by a 2 h reperfusion; (6) LY + H-Kae + I/R group, rats were injected with LY294002 (0.3 mg/kg body weight) and H-Kae (1 mg/kg body weight) 30 min prior to I/R, then subjected to a 30 min LAD coronary artery ligation followed by a 2 h reperfusion; and (7) TD + H-Kae + I/R group, rats were injected with TDZD-8 (0.3 mg/kg body weight) and H-Kae (1 mg/kg body weight) 30 min prior to I/R, then subjected to a 30 min LAD coronary artery ligation followed by a 2 h reperfusion.

### 2.6. Determination of Cardiac Function

After a 2 h reperfusion, a Philips IE33 ultrasound system (Philips Healthcare, Amsterdam, The Netherlands) was used to measure ejection fraction (EF), fractional shortening (FS), left ventricular end-diastolic pressure (LVEDP), and left ventricular systolic pressure (LVSP) of rats in the study.

### 2.7. Determination of Myocardial Infarct

To evaluate the size of I/R-related myocardial infarct in rat hearts, we used TTC staining to measure the myocardial infarct size. The experiments were performed as described previously [14, 22–24].

### 2.8. Determination of CK and LDH Activities in Serum

After a 2 h reperfusion, serum levels of CK and LDH were measured using commercial kits according to the manufacturer's instructions.

### 2.9. Assay of Oxidative Stress and Inflammatory Factors

After a 2 h reperfusion, SOD activity, MDA levels, ROS levels, CRP levels, IL-6 levels, and TNF-*α* levels were determined using commercial kits following the manufacturer's instructions.

### 2.10. TUNEL

Terminal deoxynucleotidyl transfer-mediated dUTP nick end labeling (TUNEL) was carried out using an In Situ Cell Death Detection Kit, POD (Roche, Germany) according to the manufacturer's instructions. The experiments were performed as described previously [14, 22–24].

### 2.11. Western Blot Analysis

Proteins levels of total glycogen synthase kinase-3*β* (GSK-3*β*), phospho-GSK-3*β* (P-GSK-3*β*), Akt, phospho-Akt (p-Akt), Nrf2, cleaved caspase-3, and caspase-3 were measured by Western blot. Following perfusion with the Langendorff apparatus, the same part of the rat heart was cut and collected from each sample, homogenized in buffer (50 mM Tris-HCl, pH 7.6, 0.5% Triton X-100, 20% glycerol), and centrifuged at 15,000 ×*g* for 15 min at 4°C. The supernatant was then collected and boiled for 15 min in order to denature the proteins. The resulting whole cell protein extracts were separated by electrophoresis on a 12% SDS polyacrylamide gel and subsequently transferred to nylon membrane using an electrophoretic transfer system. Membranes were then incubated first with the following primary antibodies: rabbit anti-rat GSK-3*β*, rabbit anti-rat P-GSK-3*β*, rabbit anti-rat Akt, rabbit anti-rat p-Akt, rabbit anti-rat Nrf2, rabbit anti-rat cleaved caspase-3, rabbit anti-rat caspase-3, and rabbit anti-rat *β*-actin polyclonal antibodies (Cell Signaling, Beverly, MA, USA) at 4°C overnight. Membranes were then washed with TBS-T buffer and incubated with horseradish peroxidase-conjugated secondary antibody (Cell Signaling, Beverly, MA, USA). Finally, membranes were developed using ECL-plus reagent to visualize protein bands and imaged using the Bio-Rad Gel Doc 2000 imaging system. Bio-Rad Gel Doc 2000 imaging software was used to calculate the integrated absorbance (IA) of the bands. IA = area × average density. Following normalization to *β*-actin levels, the ratios of the IAs of GSK-3*β*, P-GSK-3*β*, Akt, p-Akt, Nrf2, cleaved caspase-3, and caspase-3 to the IA of *β*-actin were used to represent relative levels of activated GSK-3*β*, P-GSK-3*β*, Akt, p-Akt, Nrf2, cleaved caspase-3, and caspase-3, respectively.

### 2.12. Statistical Analysis

Data are presented as the mean ± standard deviation from at least six independent experiments. Statistical differences were determined using analysis of variance (ANOVA), where *P* < 0.05 was considered statistically significant. The analyses were performed using the Statistical Program for Social Sciences Software (IBM SPSS, International Business Machines Corporation, Armonk City, New York, USA).

## 3. Results

### 3.1. The Effect of Kae on Cardiac Function

**Figure 1:**
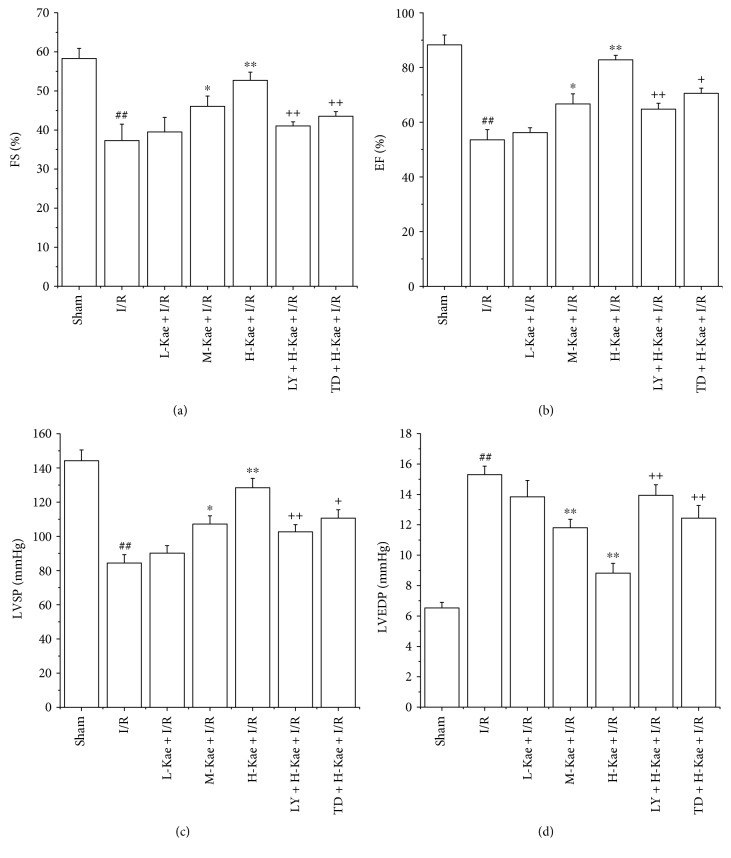
Effects of Kae treatment on cardiac function. (a) FS. (b) EF. (c) LVSP. (d) LVEDP. Values are mean ± SD (*n* = 6 in each group). ^##^*P* < 0.01 versus sham; ^∗^*P* < 0.05 and ^∗∗^*P* < 0.01 versus I/R; ^**+**^*P* < 0.05 and ^++^*P* < 0.01 versus H-Kae + I/R.

We first investigated the effect of Kae on cardiac function. As shown in Figure 1, I/R injury was found to greatly decrease FS (Figure 1(a)), EF (Figure 1(b)), and LVSP (Figure 1(c)). However, I/R injury was found to increase LVEDP (Figure 1(d)) in the I/R group (*P* < 0.01). Compared to the I/R group hearts, the M-Kae + I/R and H-Kae + I/R groups' hearts exhibited a significant functional recovery, with the efficacy of H-Kae on cardiac function found to be greater than that of M-Kae. However, this protective effect of Kae was found to be reversed by cotreatment with LY294002 or TDZD-8.

### 3.2. Kae Decreased Myocardial Injury in I/R Rats

**Figure 2:**
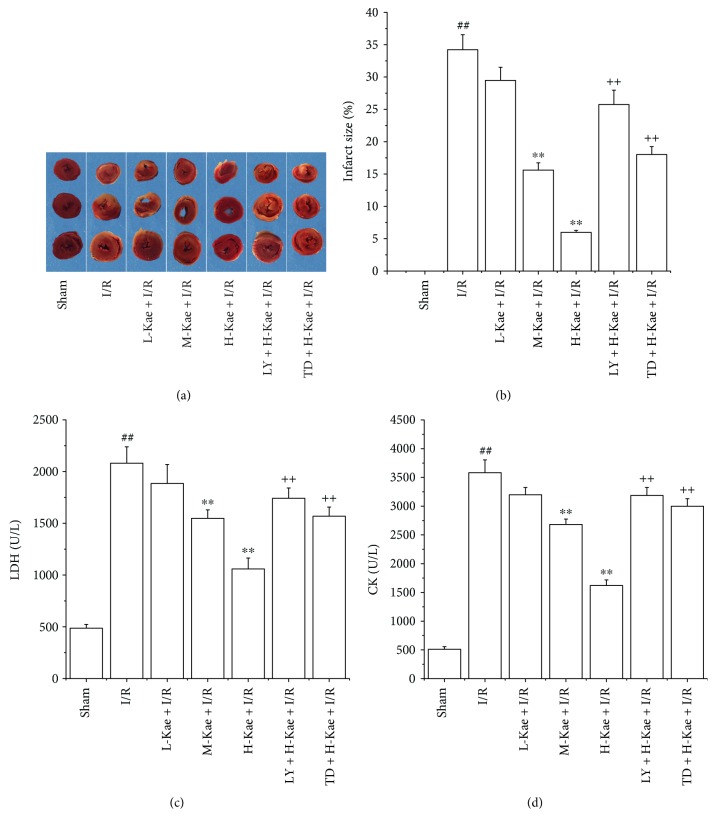
Kae treatment reduced I/R-induced myocardial injury. (a) The myocardial infarct size was measured by TTC staining. (b) The myocardial infarct size index. (c) The level of LDH. (d) The level of CK. Values are mean ± SD (*n* = 6 in each group). ^##^*P* < 0.01 versus sham; ^∗∗^*P* < 0.01 versus I/R; ^++^*P* < 0.01 versus H-Kae + I/R.

Myocardial infarct size, LDH, and CK levels were measured in order to determine whether Kae treatment resulted in a reduction in myocardial injury. We show that while hearts that underwent myocardial ischemia for 30 min followed by 2 h of reperfusion (I/R group) exhibited a significant increase in myocardial infarct size, pretreatment with M-Kae + I/R and H-Kae + I/R significantly decreased the myocardial infarct size induced by I/R, with a more significant effect observed with H-Kae compared with M-Kae (Figures 2(a) and 2(b)). Compared with the H-Kae + I/R group, pretreatment with TDZD-8 and LY294002 was found to counteract the effects of Kae on myocardial infarct size.

We demonstrate a marked increase in the leakage of myocardial isoenzyme (LDH and CK) in the I/R group after 30 min of ischemia, followed by 2 h of reperfusion, compared to that of the sham group (Figures 2(c) and 2(d)). In contrast, M-Kae and H-Kae pretreatments were found to significantly reduce the I/R-induced leakage of LDH and CK, with the inhibitory effect of H-Kae greater than that of M-Kae. However, the protective effect of Kae was demonstrated to be reversed by cotreatment with either LY294002 or TDZD-8. Considering the significant cardioprotective effects observed with Kae treatment at 1 mg/kg body weight (H-Kae), this concentration was chosen for subsequent assays.

### 3.3. Kae Protects against Myocardial I/R Injury by the Activation of PI3K/Akt/GSK-3*β* Pathways

**Figure 3:**
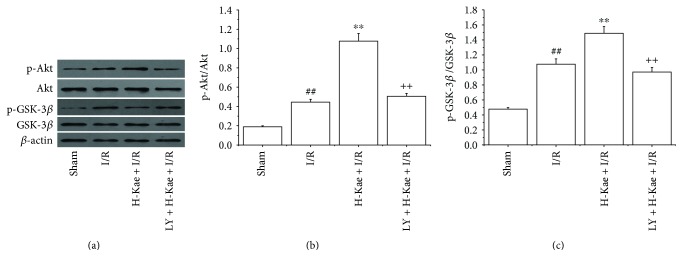
Kae treatment increased the levels of phosphorylated Akt and phosphorylated GSK-3*β* in the myocardium. PI3K inhibition (LY294002) attenuates Kae-induced increases in phospho-Akt and phospho-GSK-3*β* levels. (a) Representative Western blots for Akt, p-Akt, GSK-3*β*, and P-GSK-3*β*. (b) Grey value analysis demonstrates that Kae treatment increases the ratio of p-Akt/Akt. (c) Grey value analysis demonstrates that Kae treatment increases the ratio of p-GSK-3*β*/GSK-3*β*. ^##^*P* < 0.01 versus sham; ^∗∗^*P* < 0.01 versus I/R; ^++^*P* < 0.01 versus H-Kae + I/R.

Because the cardioprotective effects of Kae were found to be reversed by cotreatment with LY294002 (a PI3K inhibitor) or TDZD-8 (a GSK-3*β* inhibitor), we further examined the regulation of Kae on the PI3K signaling pathway. Expression levels of GSK-3*β*, p-GSK-3*β*, Akt, and p-Akt were measured using Western blot. As depicted in Figure 3, Kae treatment was found to result in a significant increase in myocardial levels of pAkt in the I/R group compared with those in the untreated I/R group. However, administration of the PI3K inhibitor, LY294002, was found to significantly attenuate the Kae-induced upregulation of p-Akt. In addition, Kae treatment was found to result in a significant increase in p-GSK-3*β* levels, a downstream kinase of Akt, in the myocardium compared with untreated I/R hearts. However, administration of the PI3K inhibitor, LY294002, was found to prevent the Kae-induced increase in GSK-3*β* phosphorylation in the I/R myocardium.

### 3.4. Kae Treatment Alleviated Oxidative Stress and Inflammation in I/R Cardiac Tissue

**Figure 4:**
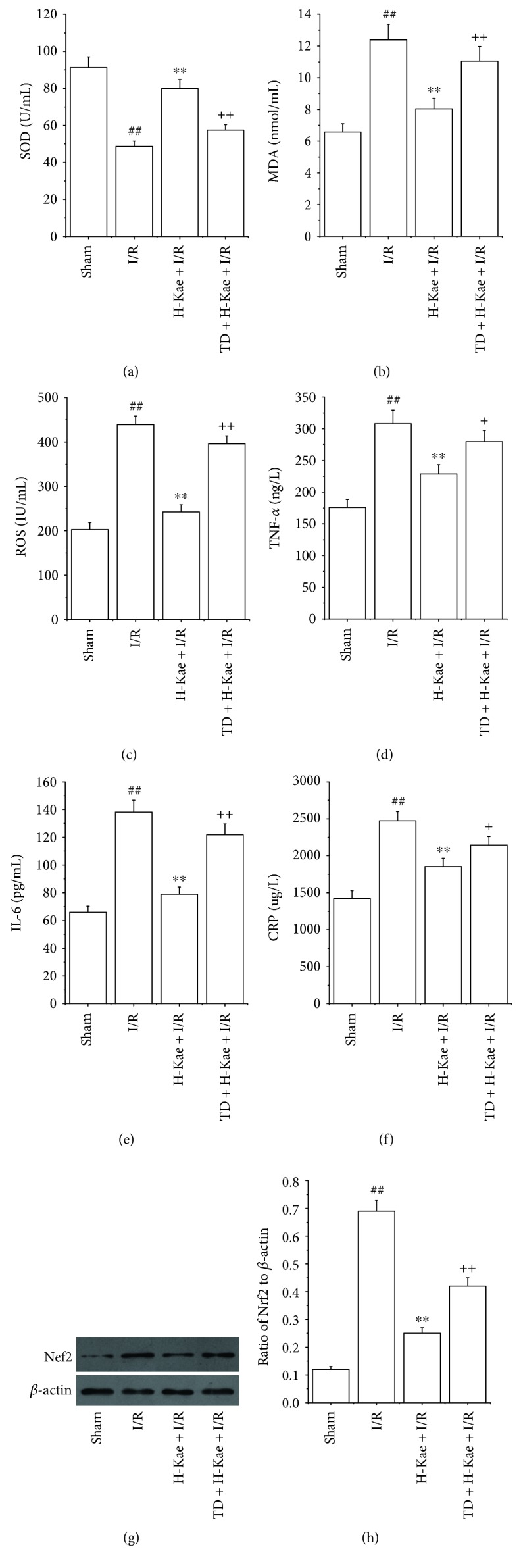
Kae preconditioning significantly suppressed oxidative stress, reduced inflammatory cytokine release, and decreased Nrf2 levels. Moreover, these effects of Kae treatment were reversed by cotreatment with TDZD-8. Effect of Kae treatment on (a) SOD, (b) MDA, (c) ROS, (d) TNF-*α*, (e) IL-6, and (f) CRP levels in I/R rat model. (g) Nrf2 protein levels in rat hearts were analyzed by Western blot. (h) Expression of Nrf2 in tissue of rat hearts by Western blot analysis (*n* = 6 in each group). ^##^*P* < 0.01 versus sham; ^∗∗^*P* < 0.01 versus I/R; ^+^*P* < 0.05 and ^++^*P* < 0.01 versus H-Kae + I/R.

Previous research has demonstrated that the overproduction of ROS, and resultant oxidative stress caused by these ROS, plays a causative role in I/R-induced cardiac injury. Thus, we carried out studies to examine the relationship between SOD activity, MDA levels, and ROS levels with I/R-induced cardiac injury. While we observed increased levels of MDA and ROS following I/R, we found that SOD activity decreased (Figures 4(a), 4(b), and 4(c)). These changes were found to be significantly inhibited with 1 *μ*g/mL Kae treatment. However, these effects of Kae on SOD, MDA, and ROS levels were found to be counteracted with TDZD-8 treatment.

Considerable evidence has demonstrated a causative role of the inflammatory response on I/R-induced cardiac injury. Interestingly, previous studies have indicated anti-inflammation properties of Kae, and effects of Kae on inflammatory serum cytokines (IL-6, TNF-*α* and CRP) were detected. Compared to the sham group, TNF-*α*, IL-6, and CRP levels were found to be significantly increased in the I/R group (*P* < 0.01) (Figures 4(d), 4(e), and 4(f)). Compared to the I/R group, the levels of these three proinflammatory cytokines were found to be significantly decreased in the H-Kae + I/R group (*P* < 0.01). However, the effects of Kae on IL-6, CRP, and TNF-*α* levels were shown to be counteracted by TDZD-8 treatment.

In order to provide insight into the mechanism of the anti-inflammation and antioxidation effects of Kae, we measured expression levels of the inflammation and oxidative stress-related protein, Nrf2, by Western blot. We show that, compared with the sham group, Nrf2 protein levels increase significantly in the I/R group (Figures 4(g) and 4(h)). In contrast with the I/R group, Nrf2 expression was found to be much lower in the H-Kae + I/R group. Interestingly, we show that GSK-3*β* inhibitor (TDZD-8) treatment was able to reverse the downregulation of Nrf2 activity induced by Kae treatment. Taken together, these results suggest that the anti-inflammatory and antioxidative effects of Kae may be regulated by Nrf2-mediated signaling pathways, which are dependent on GSK-3*β* activation.

### 3.5. Kae Suppressed Myocardial Apoptosis in I/R Cardiac Tissue

**Figure 5:**
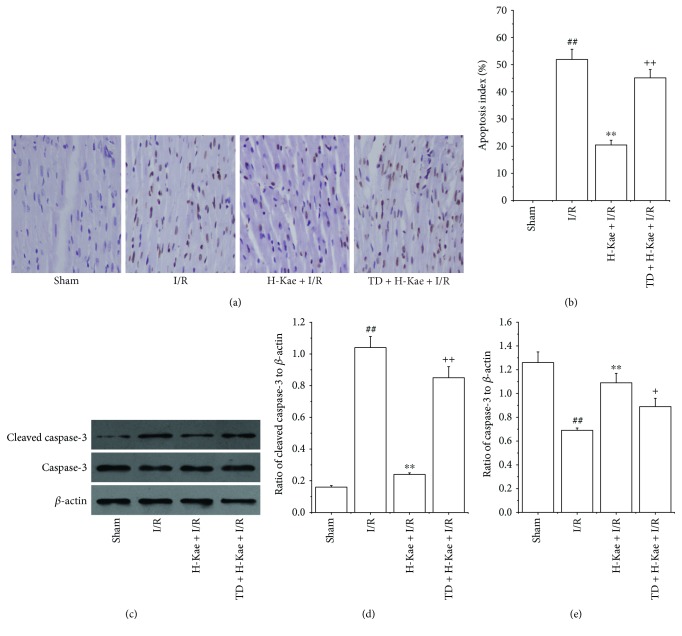
Kae preconditioning significantly inhibited cardiomyocyte apoptosis, decreased the levels of cleaved caspase-3, and increased caspase-3 levels. Moreover, these effects of Kae treatment were reversed by cotreatment with TDZD-8. (a) Suppression of cardiomyocyte apoptosis by Kae treatment (×100). Brown staining of the nucleus is indicative of apoptosis. (b) Percentage of apoptotic cells. (c) Cleaved caspase-3 and caspase-3 protein levels in rat hearts were analyzed by Western blot. Expression of cleaved caspase-3 (d) and caspase-3 (e) in tissue of rat hearts measured by Western blot analysis (*n* = 6 in each group). ^##^*P* < 0.01 versus sham; ^∗∗^*P* < 0.01 versus I/R; ^+^*P* < 0.05 and ^++^*P* < 0.01 versus H-Kae + I/R.

Apoptosis is the primary mechanism of cell death that occurs after a short period of ischemia that is followed by reperfusion. Therefore, we investigated the effects of Kae treatment on myocardial apoptosis and the apoptosis-related proteins, caspase-3, and cleaved caspase-3, in I/R cardiac tissue. We show that there is an increased activation of apoptosis in I/R cardiac tissue (Figures 5(a) and 5(b)). Compared with the I/R group, Kae treatment was found to substantially reduce this I/R-induced cardiomyocyte apoptosis. However, the antiapoptotic effect of Kae was found to be counteracted by TDZD-8 treatment. Expression levels of caspase-3 and cleaved caspase-3 were measured by Western blot (Figure 5(c)). Compared with the sham group, cleaved caspase-3 protein levels were found to be significantly increased in the I/R group, while caspase-3 protein levels were significantly decreased in the I/R group (Figures 5(d) and 5(e)). In contrast with the I/R group, cleaved caspase-3 expression was found to be much lower in the H-Kae + I/R group, while caspase-3 expression was found to be much higher in the H-Kae + I/R group. However, these effects of Kae on cleaved caspase-3 and caspase-3 levels were demonstrated to be counteracted with TDZD-8 treatment. These results suggest that the antiapoptotic effect of Kae may occur via caspase-3 pathways, which are dependent on GSK-3*β* activation.

## 4. Discussion

Myocardial I/R results in both cardiac dysfunction and myocardial infarction [25]. A number of studies have demonstrated that serum myocardial enzyme levels (CK and LDH) can be utilized to assess the degree of myocardial injury. In this study, we show that EF, FS, and LVSP levels were significantly decreased, while LVEDP levels were increased in I/R rats. In our previous studies, we found that I/R myocardium results in myocardial dysfunction, large release of enzymes (CK and LDH), myocardial infarct, and cardiomyocyte apoptosis [14, 22–24]. These results indicate that I/R results in cardiac function impairment. However, following Kae pretreatment, EF, FS, and LVSP levels were found to be significantly increased, while LVEDP levels were observed to be downregulated. This suggests that Kae treatment has the potential to improve cardiac function and protect the myocardium from I/R injury. We also observed that enzyme release (CK and LDH) and the myocardial infarct size were significantly increased following reperfusion of the ischemic myocardium. Interestingly, Kae treatment was found to significantly decrease the I/R-induced release of these enzymes, as well as the myocardial infarct size.

The PI3K/Akt signaling pathway has been demonstrated to play a critical role in the development of myocardial infarction and cardiac dysfunction following ischemia [26, 27]. Previous studies have indicated that the activation of PI3K/Akt-dependent signaling attenuates myocardial I/R injury [15, 28]. In this study, we hypothesized that activation of PI3K/Akt activity in Kae-treated rats could play a role in cardioprotection against I/R injury. We showed that phosphorylated Akt levels in the myocardium of Kae-pretreated rats were higher than those in the untreated rats. More importantly, using the PI3K inhibitor LY294002, we found that pharmacological inhibition of PI3K resulted in the abrogation of the protective effects observed with Kae. Thus, these results suggest that the potential benefits of Kae treatment in rats with myocardial I/R injury were likely mediated by the activation of PI3K/Akt pathways. GSK3*β* is a critical active enzyme that functions downstream of Akt. Phosphorylation of GSK3*β* by Akt maintains this enzyme an inactive state [29, 30]. Inactivation of GSK3*β* protects against organ ischemic injury, oxidative stress, and apoptosis [22, 31]. In the studies present here, we demonstrate elevated levels of phosphorylated GSK3*β* in the myocardium of Kae-treated rats. These elevated levels were found to correlate with reduced cardiac infarction size, decreased cardiomyocyte apoptosis, and a preservation of heart function. These results further confirm that PI3K/Akt pathways play a pivotal role in the potential therapeutic effect of Kae treatment. Moreover, we concluded that elevated myocardial PI3K/Akt signaling and the subsequent increased phosphorylation of GSK3-*β* may play important roles in the cardioprotective effects of Kae.

GSK3-*β* has been shown to function as a negative regulator of Nrf2, a protein involved in signaling pathways that plays a critical role in the defense against oxidative and toxicological stress via upregulation of antioxidant and detoxifying enzyme expression [32, 33]. In addition, Nrf2 activation has also been reported to protect against inflammation [34]. Nrf2 has been shown to attenuate inflammatory responses through a number of mechanisms, including the induction of the anti-inflammatory enzyme, hemeoxygenase-1(HO-1) [35], the negative regulation of proinflammatory cytokine expression [36], and chemokine expression [37]. In this current study, we show that Kae treatment resulted in decreased CRP, IL-6, and TNF-*α* production compared to the I/R group. Our data also indicates that Kae treatment resulted in a significant reduction in MDA and ROS levels in I/R rats, while it improved the I/R-induced reduction of total antioxidant capacity (SOD activity). These results indicate that Kae-mediated cardioprotection functions through its anti-inflammatory and antioxidation properties. Interestingly, we found that Kae treatment resulted in decreased Nrf2 protein expression levels compared to the I/R group; this is contrary to many studies (many studies suggested that activating Nrf2 may play a very important role in antioxidation and anti-inflammatory). We think that this phenomenon can be explained by the compensate effect of the body. The rats in the H-Kae + I/R group were pretreated with Kae before the LAD coronary artery ligation surgery; the extent of oxidative stress and inflammation in the H-Kae + I/R group was significantly lower than that in the I/R group, so the degree of compensate effect in the H-Kae + I/R group rats was also lower than that in the I/R group, resulting in the decrease of the protein expression of Nrf2 compared to the I/R group. However, we show that these cardioprotective effects associated with Kae treatment were blocked by treatment with the GSK-3*β* inhibitor, TDZD-8. Taken together, these results indicate that Kae-mediated cardioprotection functions through its anti-inflammatory and antioxidation properties, including its ability to activate GSK-3*β*, and thus, mediate Nrf2 signaling pathway.

Cardiomyocyte apoptosis has been shown to play a key role in the development of myocardial infarction and cardiac dysfunction following ischemia [38]. In our current study, we show that Kae treatment results in a significant reduction in myocardial apoptosis, which was consistent with the infarction size analysis. Because activation of the GSK-3*β*/caspase-3-dependent pathway has been associated with the protection of cardiomyocytes from I/R injury and inhibition of I/R-induced cardiomyocyte apoptosis [39], we hypothesized that both the activation of GSK-3*β* activity and inhibition of caspase-3 in Kae-treated rats could be responsible for the observed cardioprotection against I/R injury. We showed that phosphorylated GSK-3*β* levels in the myocardium of Kae-pretreated rats were higher than those in the untreated rats. We also showed that levels of cleaved caspase-3 in the myocardium of Kae-pretreated rats were lower than those of the untreated rats. More importantly, we found that the effect of Kae treatment on cleaved caspase-3 was reversed by cotreatment with the GSK-3*β* inhibitor, TDZD-8. Together, these results suggest that the potential benefits of Kae treatment in rats with myocardial I/R injury are likely mediated via the activation of PI3K/Akt/GSK-3*β*/caspase-3 pathways.

In conclusion, the results of our study demonstrate that Kae treatment results in the preservation of cardiac function by reducing oxidative stress, anti-inflammatory effects, myocardial infarct size, and cardiomyocyte apoptosis in the case of myocardial I/R injury. Moreover, inhibition of Nrf2 and caspase-3 via activation of the PI3K/Akt/GSK-3*β* pathway could play a critical role in the protective effects associated with Kae treatment.
